# Perspectives in educating molecular pathologists on liquid biopsy: Toward integrative, equitable, and decentralized precision oncology

**DOI:** 10.1002/1878-0261.70163

**Published:** 2025-11-14

**Authors:** Marius Ilié, Umberto Malapelle, Catherine Alix‐Panabières, Claus Lindbjerg Andersen, Sandra Chlebowski, Vivien Lake, Caroline Lacoux, Virginie Lespinet‐Fabre, Olivier Bordone, Simon Heeke, Christophe Bontoux, Ellen Heitzer, Klaus Pantel, Paul Hofman

**Affiliations:** ^1^ Laboratory of Clinical and Experimental Pathology, Hospital‐Integrated Biobank (BB‐0033‐00025), IHU RespirERA, FHU OncoAge University Hospital Centre Nice, University Côte d'Azur France; ^2^ Department of Public Health University Federico II of Naples Italy; ^3^ Laboratory of Rare Human Circulating Cells (LCCRH) and Liquid Biopsy University Medical Centre of Montpellier France; ^4^ CREEC (CREES) Unité Mixte de Recherches, IRD 224–CNRS 5290–Université de Montpellier France; ^5^ European Liquid Biopsy Society (ELBS) Hamburg Germany; ^6^ Department of Molecular Medicine Aarhus University Hospital Denmark; ^7^ Department of Clinical Medicine Aarhus University Denmark; ^8^ The Danish National Center for Circulating Tumor DNA Guided Cancer Treatment Aarhus Denmark; ^9^ University Côte d'Azur Initiative of Excellence Nice France; ^10^ Center for Active Learning and MSc International Office IDEX Université Côte d'Azur Nice France; ^11^ Department of Thoracic/Head & Neck Medical Oncology The University of Texas MD Anderson Cancer Center Houston TX USA; ^12^ Department of Pathology Cancer University Institute of Toulouse‐Oncopole France; ^13^ Institute of Human Genetics, Diagnostic and Research Center for Molecular BioMedicine Medical University of Graz Austria; ^14^ Department of Tumor Biology University Medical, Center Hamburg‐Eppendorf Germany

**Keywords:** cfDNA, CTC, ctDNA, education, EMMP, liquid biopsy, molecular pathology

## Abstract

Liquid biopsy has transformed molecular oncology by enabling noninvasive, real‐time monitoring of cancer progression, treatment responses, and detection of minimal residual disease. Despite technological advances, educational gaps, regarding standardized, comprehensive training for molecular pathologists, remain. To address these, a new educational program, the European Masters in Molecular Pathology (EMMP) developed a dedicated educational module aimed at providing pathologists with specialized competencies in liquid biopsy. In this perspective, we discuss how embedding liquid biopsy training within the integrative pathology framework, linking molecular diagnostics, histopathological findings, and clinical context, enhances diagnostic accuracy and therapeutic decision‐making in the clinic. Furthermore, we emphasize the importance of decentralizing liquid biopsy expertise to local pathology units, reducing dependency on external commercial platforms, ensuring data sovereignty, and enabling rapid, cost‐effective diagnostics. Finally, integrating health policy and ethical considerations within liquid biopsy education prepares future molecular pathologists to engage meaningfully in shaping policy frameworks that support equitable and sustainable clinical implementation of precision oncology. In summary, we propose the EMMP module as a comprehensive educational strategy for training molecular pathologists in the latest liquid biopsy technologies and advancements.

## Graphical abstract

Liquid biopsy enables minimally invasive, real‐time molecular profiling through analysis of circulating biomarkers in biological fluids. This Perspective highlights the importance of training pathologists through integrative educational programs, such as the European Masters in Molecular Pathology, to ensure effective and equitable implementation of liquid biopsy in precision oncology.

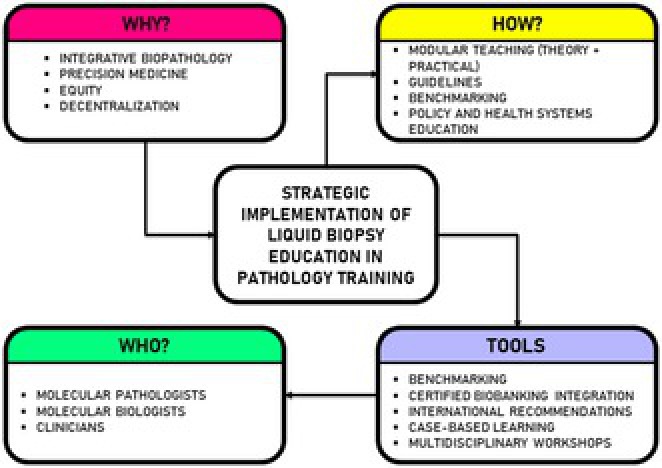

**Abbreviations**
CAP: College of American Pathologists
cfDNA: circulating cell‐free DNA
CTCs: circulating tumor cells
ctDNA: circulating tumor DNA
ELBS: European Liquid Biopsy Society
EMMP: European Masters in Molecular Pathology
ESP: European Society of Pathology
IASLC: International Association for the Study of Lung Cancer
ISLB: International Society of Liquid Biopsy
ISMRC: International Symposium on Minimal Residual Cancer
NGS: next‐generation sequencing

## 1 Introduction

In the era of precision oncology, the demand for minimally invasive, real‐time tools to characterize tumor biology has driven the development and integration of liquid biopsy into clinical practice. Liquid biopsy refers to the minimally invasive sampling and analysis of biological fluids (primarily blood, but also, pleural effusion, ascites, urine, saliva, cerebrospinal fluid, etc.) to detect circulating biomarkers, such as circulating tumor cells (CTCs), circulating cell‐free DNA (cfDNA), circulating tumor DNA (ctDNA), extracellular vesicles, plasma proteins, and circulating RNAs (miRNAs, lncRNAs), particularly derived from tumor lesions [1, 2]. This innovative approach has dramatically transformed molecular testing for targeted therapies, real‐time monitoring of molecular hallmarks and dynamics, early detection, tracking minimal residual disease (MRD), assessing treatment response, and understanding tumor evolution and heterogeneity [3, 4, 5, 6].

In clinical practice, the significance of liquid biopsy is pivotal because it overcomes several limitations inherent in tissue biopsies, including invasiveness, tumor accessibility, tumor heterogeneity, repeatability and cost‐effectiveness. Liquid biopsy dynamically provides a comprehensive snapshot of the genomic landscape of cancers, allowing for the detection of actionable mutations, monitoring of clonal evolution, and making informed clinical decisions in precision oncology [7]. Importantly, this approach has been rapidly integrated into international guidelines for clinically managing cancer patients, including lung, colorectal, breast and prostate cancer, particularly those published by major oncology societies, such as NCCN and ESMO [8, 9, 10, 11].

Moreover, liquid biopsy benefits from the easy collection of longitudinal samples, thus enabling continuous monitoring of disease progression and treatment efficacy, personalizing clinical management and fostering the emergence of dynamic biomarkers [12, 13, 14]. As molecular pathology increasingly relies on genomic profiling for precision therapy, the use of liquid biopsy has become critical for comprehensive patient assessment complementary to tissue‐based molecular analysis [15].

Nevertheless, the widespread implementation of liquid biopsy still faces many challenges, including standardization of preanalytical procedures, variability in assay sensitivity, especially in early‐stage disease, and the need for robust clinical validation across tumor types. Finally, cost and reimbursement of molecular testing from liquid biopsy is certainly a major bottleneck for using liquid biopsy in daily practice. Addressing these hurdles will be key to ensuring its full integration into routine clinical care.

This perspective article examines the current state and gaps in liquid biopsy education, offering an expert view informed by the experiences of the European Master's degree in Molecular Pathology (EMMP; https://univ‐cotedazur.eu/msc/european‐msc‐molecular‐pathology), and proposes strategies for effective educational models to equip pathologists to implement liquid biopsy comprehensively in clinical practice.

## 2 Integrative pathology in liquid biopsy education

The rapid advancement of liquid biopsy in clinical oncology necessitates a fundamental transformation in the conceptual approach to pathology education [16, 17]. While traditional pathology has primarily focused on tissue and cytology‐based diagnostics, modern precision medicine demands a more integrated diagnostic framework. Integrative pathology embodies this approach by combining molecular diagnostics, histopathological evaluation, and clinical context into a cohesive diagnostic interpretation. This theoretical integration not only facilitates a more comprehensive understanding of disease processes but also enhances diagnostic accuracy and clinical decision‐making [18]. Contemporary pathologists must therefore develop expertise beyond traditional histomorphology, gaining proficiency in molecular biology, genomics, bioinformatics, and especially in the principles and practice of liquid biopsy. Rather than replacing conventional tissue biopsies, integrative pathology positions liquid biopsy as complementary, providing critical molecular insights particularly when traditional biopsies are limited or insufficient for comprehensive genomic profiling. This integration enables pathologists to interpret liquid biopsy data within the broader context of morphological and clinical findings, thus facilitating a holistic approach to patient care [6, 7].

Recognizing these evolving demands, professional organizations, such as the European Society of Pathology (ESP) and the College of American Pathologists (CAP) have advocated explicitly for the integration of molecular diagnostics into pathology curricula, underscoring molecular pathology as an essential element of modern cancer diagnosis and management [19, 20, 21]. In practice, this advocacy is increasingly reflected in structured initiatives. For example, ESP has introduced dedicated molecular pathology courses that include liquid biopsy sessions, integrating molecular data interpretation with histopathological case review. Similarly, the CAP–ASCO joint guidelines on ctDNA testing have been incorporated into training resources, providing practical pathways for embedding liquid biopsy into clinical workflows. Beyond guidelines, the International Association for the Study of Lung Cancer (IASLC) organizes educational workshops where liquid biopsy case studies are taught alongside morphology and imaging, illustrating integrative diagnostics in lung cancer.

Similarly, major oncology organizations, including ESMO, ASCO, AACR, and EACR, increasingly integrate liquid biopsy into their scientific agendas and clinical practice guidelines, reinforcing the critical role of integrative pathology within the current landscape of precision oncology. By combining conceptual frameworks with these concrete training initiatives, integrative pathology emerges as both a theoretical imperative and a practical educational paradigm. Embedding liquid biopsy within this framework prepares future pathologists to translate molecular insights into actionable clinical decisions, ultimately strengthening the role of pathology in precision oncology.

## 3 Toward decentralization: Empowering local pathology units

Centralized diagnostic models that rely heavily on external commercial providers can result in inefficiencies, delayed patient care, and diminished data control. To address these challenges, educational programs should prioritize decentralization strategies, aiming to strengthen the molecular diagnostic capabilities of local pathology units. Training local professionals, especially pathologists, in comprehensive liquid biopsy workflows enables healthcare facilities to maintain critical diagnostic expertise internally, significantly reducing reliance on external laboratories [22, 23, 24].

Decentralization has multiple tangible advantages. Firstly, it safeguards data sovereignty by keeping sensitive patient information within local healthcare institutions, enhancing both privacy and security. Secondly, localizing diagnostic capabilities markedly improves diagnostic agility, allowing rapid adaptation of testing protocols to meet evolving clinical demands. This translates into reduced turnaround times and quicker therapeutic decisions, thereby enhancing patient outcomes [22, 23, 24]. Thirdly, retaining diagnostic expertise locally can improve overall cost‐effectiveness by reducing outsourcing expenditures, optimizing resource utilization, and minimizing logistical complexities associated with external commercial testing.

Furthermore, decentralized diagnostic infrastructures can contribute indirectly but significantly to equitable healthcare access. By ensuring advanced molecular diagnostic capabilities are more widely distributed and not confined to centralized facilities, decentralization helps level disparities between well‐resourced and underserved regions. Although detailed considerations of equity will be discussed in the subsequent section, it is important to recognize here that decentralization lays essential groundwork for equitable distribution of precision oncology services.

Educational initiatives like the EMMP must therefore prioritize training pathologists and technical personnel not only to master advanced molecular techniques but also to become proficient in independently managing liquid biopsy workflows within their respective institutions. By cultivating local expertise, pathology units can sustainably deliver precise and responsive molecular diagnostics, reinforcing both clinical efficiency and broader healthcare equity.

## 4 Equitable access: Liquid biopsy as a tool for global health equity

Significant global disparities persist in access to advanced diagnostic technologies, particularly predictive biomarker testing, underscoring the urgent need to address equity in healthcare delivery [25]. These disparities are rooted not only in socioeconomic inequalities and limited resources, but also in systemic barriers, such as uneven regulatory approvals for liquid biopsy assays and the high cost of consumables—including extraction kits, sequencing reagents, and data analysis pipelines—which place additional burdens on low‐ and middle‐income healthcare systems. Liquid biopsy, as a minimally invasive, scalable, and repeatable method, represents a uniquely powerful approach to mitigate these inequities by broadening access to essential molecular diagnostics across diverse healthcare settings [26]. To leverage liquid biopsy effectively in achieving global equity, structured educational initiatives must specifically target pathologists and laboratory professionals worldwide.

Such programs should explicitly emphasize strategies for overcoming geographic and economic barriers, while also raising awareness of how reimbursement frameworks, regulatory heterogeneity, and consumable costs affect implementation. Educational curricula must therefore prioritize both technical competence and an awareness of the socioeconomic contexts influencing diagnostic access, thereby democratizing precision oncology on a global scale [27].

Within this context, initiatives such as the EMMP Liquid Biopsy Module prepare future molecular pathologists not only by delivering technical training but also by embedding awareness of policy, sustainability, and cost‐effectiveness. This curriculum highlights benchmarking of different technologies to identify affordable yet reliable solutions, promotes collaborations with industry to facilitate access to validated platforms, and incorporates discussions on engaging regulators to harmonize approval pathways. In doing so, it equips trainees with both the technical competencies and the advocacy tools necessary to advance equitable molecular diagnostics.

Decentralization strategies, as previously discussed, play a complementary role in promoting equity by fostering local diagnostic autonomy. However, overcoming systemic inequities requires broader structural reforms, including regulatory alignment, sustainable financing, and strategic partnerships between academia, healthcare institutions, and policymakers [23].

Despite much progress, several gaps remain in practical molecular diagnostic training within pathology curricula, especially in resource‐limited regions. Addressing these deficits through structured and focused programs like EMMP is pivotal to ensuring not only equitable adoption of liquid biopsy worldwide but also its sustainable and scalable integration into precision oncology.

## 5 International standardization and the role of professional societies

Several international professional societies, prominently the International Society of Liquid Biopsy (ISLB) and the European Liquid Biopsy Society (ELBS), actively lead global initiatives aimed at the standardization and clinical implementation of liquid biopsy practices [28, 29]. Both organizations are critical to defining international standards, harmonizing laboratory methodologies, and ensuring the uniformity and clinical validity of liquid biopsy assays through the development of expert guidelines and consensus recommendations [28, 29, 30, 31, 32].

The ISLB organizes major international meetings, including the ISLB Annual Meeting, providing a global forum for education, exchange, and guideline dissemination. The International Symposium on Minimal Residual Cancer (ISMRC) is organized thanks to the ELBS. In addition, the ACTC meetings, organized by the Hellenic Society of Liquid Biopsy (www.actc2025.org) have been held seven times over the past decade. These gatherings serve as key platforms for education, interdisciplinary dialogue, and collaborative innovation, facilitating global exchange among researchers, clinicians, and pathologists. Similarly, the ELBS provides structured training courses, workshops, and expert‐authored guidelines specifically designed to standardize liquid biopsy methodologies across European institutions and beyond. By providing clearly defined minimum testing standards, these societies directly ensure consistency and reliability in clinical diagnostics [28, 29, 30, 31, 32].

Despite these structured efforts, recent surveys indicate that a significant number of practicing pathologists still feel inadequately prepared to implement liquid biopsy routinely, highlighting the ongoing shortfall in standardized educational pathways [33, 34, 35].

These gaps further reinforce the necessity of comprehensive educational initiatives that encompass not only technical proficiency but also training in ethical, legal, regulatory, and quality management frameworks. Through such inclusive instruction, pathologists can become fully prepared to responsibly integrate liquid biopsy within precision oncology workflows [36, 37, 38].

Other international organizations have joined ISLB and ELBS in promoting structured liquid biopsy education and standardization (Table 1):

- The American Society of Clinical Oncology (ASCO) and the College of American Pathologists (CAP) collaborate extensively on training programs and publish detailed guidelines on circulating tumor DNA (ctDNA) testing, aiming to standardize clinical liquid biopsy implementation within the United States [18].
- The IASLC provides specialized training in liquid biopsy for lung cancer pathologists and clinicians globally, fostering standardized and clinically valid diagnostic practices [11].
- The MD Anderson Cancer Center (Houston, TX, USA) offers specialized fellowship programs in molecular diagnostics and personalized oncology, explicitly incorporating liquid biopsy training into their curriculum. These fellowships have set international benchmarks for practical molecular pathology education [39, 40].
- The Danish National Centre for Circulating Tumor DNA‐Guided Cancer Treatment supports practical, real‐world training through workshops within clinical trial frameworks, enabling pathologists and researchers to confidently integrate liquid biopsy diagnostics into routine clinical settings [41, 42].

**Table 1:** Educational framework and international outreach of the liquid biopsy module within the European Masters in Molecular Pathology.

(A) Geographic distribution of major liquid biopsy training initiatives.	
International liquid biopsy training programs initiatives	Focus area
EMMP (Europe)	Comprehensive academic training in molecular pathology and liquid biopsy
ELBS Education program (Global)	Standardization of preanalytical, analytical, and data interpretation protocols; Clinical validation and support for regulatory approval of LB technologies; Education, dissemination and training programs for healthcare professionals and scientists; Research innovation through collaborative studies and biomarker discovery; Equitable access and patient‐centered implementation across Europe with a bridge with America, Asia, and Australia.
ISLB Education Program (Global)	Standardized workshops, webinars, manuals, and guidelines on LB methodologies
ASCO/CAP Joint Guidelines (USA)	Clinical guidelines and implementation support for ctDNA
IASLC Training Programs (Global)	Workshops on lung cancer LB applications
MD Anderson Fellowships (USA)	Fellowships in molecular diagnostics, liquid biopsy, and personalized medicine
Danish National ctDNA Center (Denmark)	Training within national ctDNA‐driven clinical trials

(B) EMMP liquid biopsy module overview.	
Module component	Key features
Foundations	Introduction to liquid biopsy and molecular pathology
Technical training	cfDNA/ctDNA/CTC analysis, NGS, digital PCR
Clinical relevance	Case‐based learning across multiple cancer types
Emerging innovations	MRD, nonblood biofluids, cutting‐edge platforms
Interactive learning	Debates, group work, hands‐on lab sessions
Assessment and outcomes	Skills evaluation, knowledge testing, practical competence

Collectively, these international initiatives underscore a clear global consensus on the necessity of structured, interdisciplinary educational programs in liquid biopsy. The EMMP's Liquid Biopsy Module explicitly incorporates these international standards and expert recommendations into its educational framework, thereby serving as a practical benchmark for training. This close alignment ensures EMMP graduates develop competencies fully consistent with internationally recognized best practices, positioning them effectively to advance precision oncology through expert liquid biopsy implementation and clinical innovation.

## 6 Incorporating health policy and ethical dimensions

Effective education in liquid biopsy must incorporate comprehensive training in healthcare policy and ethical frameworks, recognizing that the successful clinical implementation of liquid biopsy technologies extends beyond purely technical competence. Pathologists and laboratory professionals should gain a clear understanding of diverse policy dimensions, including reimbursement structures, regulatory pathways, patient data privacy, and equity considerations, as these directly influence the availability and accessibility of molecular diagnostics [43, 44, 45].

In particular, understanding reimbursement policies is essential. Trainees should become familiar with healthcare system‐specific reimbursement models, recognizing how variations in coverage and compensation for liquid biopsy tests can significantly impact patient access and clinical adoption. Similarly, regulatory knowledge is indispensable; trainees must understand the approval processes for new diagnostic tests, the roles of regulatory bodies, and how to navigate evolving regulatory landscapes to ensure compliance and timely implementation.

Ethical considerations, such as patient privacy, informed consent, and secure data handling, are similarly emphasized to ensure responsible and transparent liquid biopsy implementation. Educational programs must emphasize responsible handling of genomic data, clarifying trainees' ethical obligations to protect patient confidentiality and ensure transparency throughout testing procedures [43, 44, 45].

A critical policy dimension is the active advocacy required to ensure equitable patient access to precision oncology. Robust policy frameworks are essential to ensure the equitable distribution and effective integration of advanced diagnostic technologies across diverse populations and healthcare systems [43, 44, 45]. Specifically, Horgan *et al*. advocate for strategies to democratize clinical trials and genomic diagnostics, emphasizing the ethical and policy implications of broadening access to liquid biopsy technologies, particularly in resource‐limited environments [43, 44, 45].

To deliver these components effectively, the module integrates health policy and ethics into the curriculum through interactive seminars, expert‐led workshops, and case‐based discussions. For example, seminars introduce students to reimbursement models and regulatory pathways across Europe and internationally; case discussions explore real‐world issues, such as patient consent for cfDNA testing, incidental findings, and compliance with General Data Protection Regulation (GDPR); and workshops led by health policy experts and ethicists provide practical guidance on navigating legal and ethical frameworks. Structured debates further encourage students to critically evaluate policy challenges and develop advocacy strategies.

By embedding these health policy and ethical dimensions into educational curricula, pathologists become better equipped to engage proactively with policymakers, administrators, and patient advocacy groups. This comprehensive training ensures that molecular pathologists do not merely implement technologies passively but become informed advocates capable of influencing policy decisions, ultimately promoting fair, inclusive, and sustainable precision oncology practices globally.

## 7 Education methodology: A practical and integrated framework

Building on the preceding discussion of global standardization and integrative pathology, this section outlines how these principles are operationalized through the European Masters in Molecular Pathology (EMMP) Liquid Biopsy (LB) Module. The program addresses critical educational gaps by providing a comprehensive curriculum that combines theoretical instruction, hands‐on technical training, and clinical case integration [20]. The module begins with a strong conceptual foundation, emphasizing the clinical relevance of LB and the biological and diagnostic importance of key circulating biomarkers, such as cfDNA, ctDNA, CTCs, extracellular vesicles, and noncoding RNAs [20, 46]. By linking molecular data to histopathology and clinical context, the EMMP promotes an integrative diagnostic mindset essential for precision oncology [47].

Students then progress to practical laboratory workshops under international experts, acquiring core technical skills in cfDNA extraction, next‐generation sequencing (NGS), digital PCR, RT‐PCR, and CTC enrichment [20, 46]. Emphasis is placed on standardized preanalytical workflows, blood collection, plasma processing, nucleic acid extraction, and sample storage, to ensure accuracy and reproducibility in clinical diagnostics.

Case‐based discussions and structured debates anchor the clinical application of LB. Students analyze real oncology cases, integrating genomic data with histopathological findings to inform diagnosis, therapy selection, and disease monitoring [48]. These interactive sessions cultivate critical thinking and communication skills, preparing students for multidisciplinary team‐based practice.

To ensure adaptability in a rapidly evolving field, trainees are also introduced to emerging innovations, including hybrid capture sequencing, comprehensive genomic profiling, and the analysis of alternative biofluids (pleural effusion, cerebrospinal fluid, bone marrow, sputum, and urine), which demonstrate LB's expanding clinical scope [49, 50, 51, 52].

Student performance is assessed through a mix of practical evaluations, written assignments, and literature‐based analyses, ensuring both technical mastery and conceptual depth. This multidimensional framework enables EMMP graduates to effectively apply LB technologies in diagnostics and translational research, embodying the principles of integrative pathology and global equity emphasized throughout this Perspective (Table 1) [20].

## 8 Limitations and future perspectives

Despite the comprehensive educational structure and substantial benefits offered by the Liquid Biopsy Module within the EMMP, certain limitations remain that warrant consideration and continuous improvement in the field of liquid biopsies. The accelerated pace of innovation in genomics and molecular diagnostics presents a challenge to maintaining up‐to‐date course content [3]. Ensuring that the module reflects the latest developments requires regular updates and a sustained commitment from faculty to integrate emerging research, novel biomarkers, and cutting‐edge technologies into teaching.

**Fig. 1:**
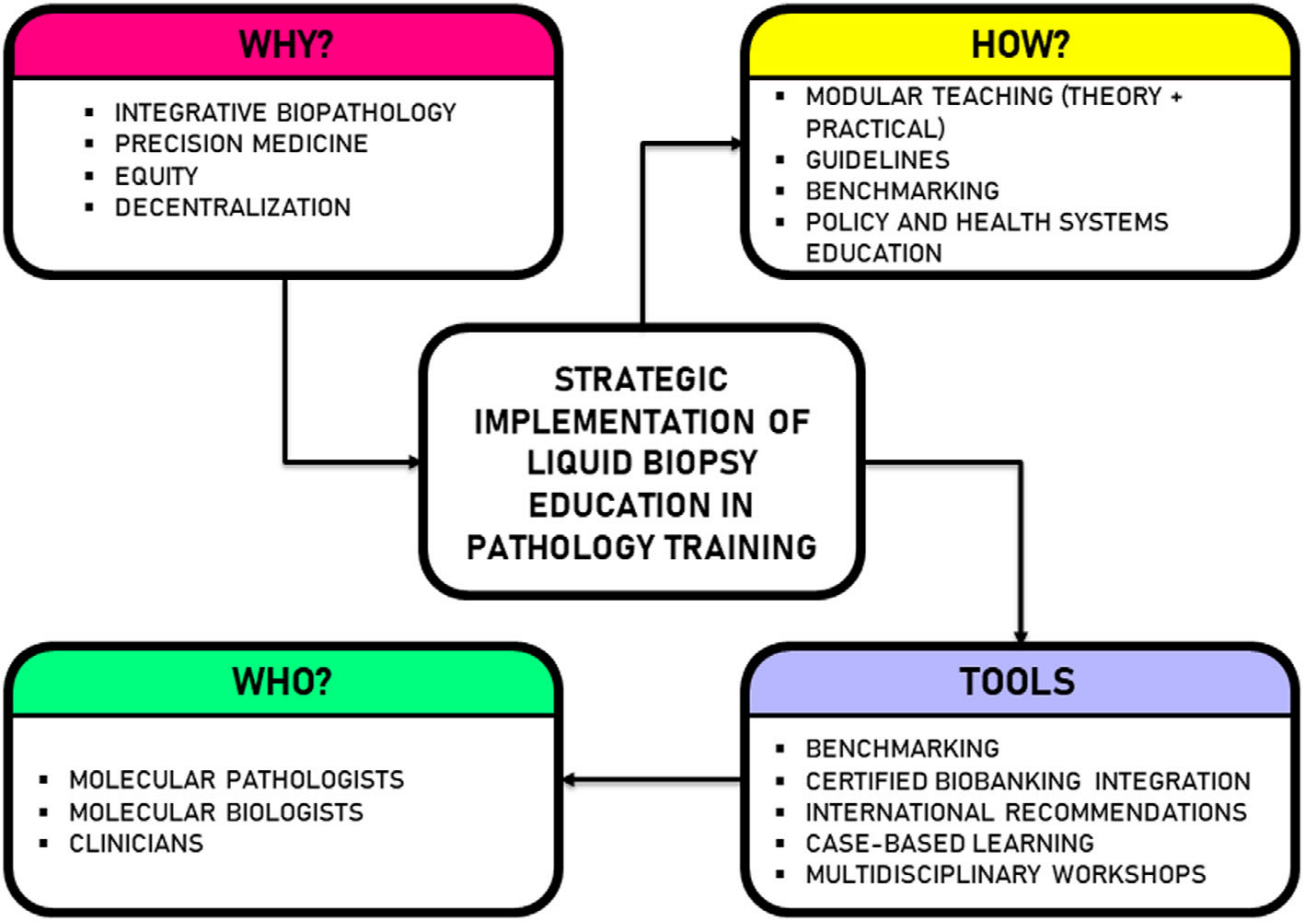
Strategic implementation of liquid biopsy education in pathology training.

Another limitation is the relatively condensed duration of the module, which may constrain the depth and breadth of certain topics, particularly complex bioinformatics analyses, which require extensive practical experience and training. Additionally, students may enter the module with varying levels of prior experience in molecular techniques, potentially affecting their initial ability to fully benefit from advanced practical sessions. This variability could be mitigated by offering preparatory materials or introductory online courses to ensure a more level starting point and improve overall learning outcomes (Fig. 1).

Furthermore, the practical workshops, although extensive, might not replicate the complexity of clinical settings where high sample throughput, quality assurance constraints, and varied clinical conditions are encountered. Thus, while students gain valuable laboratory skills, additional real‐world clinical laboratory internships or extended practical placements may be beneficial for comprehensive training (Fig. 1).

Looking toward future perspectives, this module holds significant potential for growth and innovation. Expansion of collaborative efforts with clinical institutions and biotechnology companies could provide students greater exposure to advanced analytical platforms, novel biomarkers, and ongoing clinical trials (Fig. 1). Partnerships could also offer opportunities for internships, facilitating real‐world experience and ensuring students are better prepared for immediate professional integration.

Teaching should include the ability to benchmark various technical solutions (preanalytics, analytical platforms, and informatics pipelines). Training must also foster integration with certified biobanking infrastructures, ensuring sample traceability, harmonization, and quality assurance (Fig. 1).

The pedagogical strategy should incorporate a health policy dimension, preparing pathologists to understand the broader implications of liquid biopsy technologies within healthcare systems and public policy frameworks [44]. Trainees must become aware of the policies shaping the implementation and accessibility of precision oncology tools, such as reimbursement models, regulatory approvals, data privacy, and equity in healthcare delivery [43, 45]. Understanding these aspects helps pathologists advocate for equitable patient access to liquid biopsy testing, ensuring that innovations translate effectively from research into clinical practice.

Furthermore, enhancing the bioinformatics component of the module by including specialized training in data analytics, artificial intelligence, and machine learning techniques for liquid biopsy data interpretation could significantly enrich the module's applicability. Integrating digital resources, such as interactive virtual laboratories, could offer additional flexibility and reinforce learning outcomes.

Lastly, developing an alumni network and a continuing education framework could ensure sustained learning and professional development, allowing graduates to remain updated on advancements in liquid biopsy methodologies and best practices. Through these targeted enhancements, the Liquid Biopsy Module can foster highly skilled professionals capable of driving future innovations in precision oncology.

## 9 Future perspectives

The Liquid Biopsy Module of the EMMP represents a pioneering educational initiative that directly responds to the evolving demands of molecular pathology and precision oncology. By combining rigorous theoretical instruction with extensive hands‐on experience, the module equips future pathologists with the scientific and technical expertise required to effectively implement liquid biopsy technologies in both clinical diagnostics and translational research.

Through a multidisciplinary and integrative approach, students engage in interactive lectures, laboratory workshops, and clinical case discussions guided by international experts. These activities cultivate critical thinking, diagnostic accuracy, and the ability to interpret complex molecular data—skills essential for the next generation of molecular pathologists.

While challenges remain, such as adapting curricula to technological advancements and expanding practical exposure, the EMMP actively addresses these through continuous updates, partnerships with clinical institutions and industry, and the integration of advanced bioinformatics training. Moving forward, collaboration with biotechnology companies and research consortia will enhance access to emerging analytical platforms and foster real‐world learning opportunities.

Ultimately, the EMMP aims to serve as a model for international training in liquid biopsy, setting a global benchmark for molecular pathology education. By empowering pathologists to translate innovative diagnostic tools into improved patient outcomes, the program contributes meaningfully to the broader implementation and standardization of liquid biopsy within precision oncology worldwide.

## Conflict of interest

The authors declare no conflict of interest.

## Author contributions

MI and PH conceived and designed the project. MI, UM, CAP, CLA, SC, VL, CL, VLF, OB, SH, CB, EH, KP, and PH acquired the data. MI and PH wrote the paper. All authors reviewed the paper.
